# Models of Psychedelic-Assisted Psychotherapy: A Contemporary Assessment and an Introduction to EMBARK, a Transdiagnostic, Trans-Drug Model

**DOI:** 10.3389/fpsyg.2022.866018

**Published:** 2022-06-02

**Authors:** William Brennan, Alexander B. Belser

**Affiliations:** ^1^Cybin, Inc., Toronto, ON, Canada; ^2^Fordham University, New York City, NY, United States

**Keywords:** psychedelic assisted therapy, psychotherapy models, therapist training, research utilization, psychedelic drugs, psychedelic drug use

## Abstract

The current standard of care in most uses of psychedelic medicines for the treatment of psychiatric indications includes the provision of a supportive therapeutic context before, during, and after drug administration. A diversity of psychedelic-assisted psychotherapy (PAP) models has been created to meet this need. The current article briefly reviews the strengths and limitations of these models, which are divided into basic support models and EBT-inclusive therapy models. It then discusses several shortcomings both types of models share, including a lack of adequate attention to embodied and relational elements of treatment, and insufficient attention to ethical concerns. The article then introduces the EMBARK model, a transdiagnostic, trans-drug framework for the provision of supportive psychotherapy in PAP clinical trials and the training of study therapists. EMBARK was designed to overcome challenges that prior models have had in conceptualizing therapeutic change in psychedelic treatment, incorporating elements of non-psychedelic evidence-based therapies, incorporating therapists’ prior skills and clinical orientations, delimiting therapist interventions for research standardization, and determining specific factors that contribute to treatment outcomes. The article explains EMBARK’s six clinical domains, which represent parallel conceptualizations of how therapists may support therapeutic benefit in PAP treatment, and its four care cornerstones, which reflect therapists’ broad ethical responsibility to participants. The article describes how these elements of the model come together to structure and inform therapeutic interventions during preparation, medicine, and integration sessions. Additionally, the article will discuss how EMBARK therapist training is organized and conducted. Finally, it will demonstrate the broad applicability of EMBARK by describing several current and upcoming PAP clinical trials that have adopted it as the therapeutic frame.

## Introduction

Psychedelic medicines are a distinctive class of pharmacotherapeutic interventions. Perhaps their main distinguishing feature is that a significant portion of their purported efficacy in reducing psychiatric symptoms is thought to derive, not from their direct biochemical effects on the brain, but from their capacity to foster acute and subacute shifts in a participant’s subjective experience that, if skillfully handled, can result in beneficial outcomes (Oram, 2014). As such, all but one of the registered clinical trials of “classic” (Johnson et al., 2019), serotonergic psychedelic drugs being used to treat a mental health condition have couched drug administration within a context of drug-appropriate psychotherapy (Reiff et al., 2020). Accordingly, MDMA, a quasi-psychedelic drug (Nichols, 1986) whose models of clinical application closely match those of classic psychedelics, is expected to become the first FDA-approved drug for which the administration protocol mandates a psychotherapeutic frame (Emerson and Cooper, 2021; Servick, 2021). Although some actors in the field are seeking to develop drugs that replicate the putatively therapeutic neuropharmacology of classic psychedelics while eliminating their subjective effects (Dong et al., 2021)—and thereby the need for supportive therapy (Yakowicz, 2021)—the potential efficacy of this approach remains speculative (Yaden and Griffiths, 2020; Olson, 2021). In fact, it may be more likely that the widely touted increases in neuroplasticity induced by some psychedelics reflect a pluripotent state that requires therapeutic framing to bring about enduring beneficial outcomes (Vollenweider and Kometer, 2010; Carhart-Harris et al., 2018a; Dölen, 2021; Lepow et al., 2021). As such, the first wave of psychedelic medicines in psychiatry will almost certainly hold as axiomatic that psychedelic medicines are best administered within a well-considered context of psychotherapy.

A diversity of adjunctive psychotherapy models has arisen to provide such a context (Horton et al., 2021). They have been developed by clinical research groups within academic, corporate, and non-profit entities to support their trials of various psychedelic medicines in the treatment of a range of indications. The specific approaches of these models have drawn influence from the work of early PAP pioneers inside and outside of formal research contexts (Eisner, 1967; Grof, 1980; Greer and Tolbert, 1986; Stolaroff, 2004), indigenous approaches to the use of psychedelic substances for healing (Fotiou, 2020), and elements of non-psychedelic therapeutic approaches (Walsh and Thiessen, 2018; Horton et al., 2021). Although these models share some similarities (e.g., attention to set and setting, a structure consisting of preparation, medicine, and integration sessions), they vary greatly in how much non-drug therapy time they offer; the extent to which they incorporate extrinsic, non-psychedelic psychotherapeutic knowledge and best practices; and whether they view the support they provide as therapy *per se* or a distinct form of non-psychotherapeutic support. These differences are to be expected given the dearth of research that examines which, if any, of these factors impact treatment efficacy. This article presents a speculative assessment of the strengths and shortcomings of these models based on a review of the available empirical literature on psychedelic-assisted psychotherapy (PAP). For its purposes, it will focus on clinical trials treating specific indications for which the results and/or therapeutic approach have been presented in peer-reviewed publications and that have examined the efficacy of long-acting classic psychedelics (e.g., psilocybin, ayahuasca, LSD) or MDMA due to strong similarities in the therapeutic framing of these drugs. It will omit ketamine and short-acting psychedelics (e.g., 5-MeO-DMT) due to the different considerations for therapeutic framing that they present.

The current article also introduces the EMBARK model of PAP, which grew out of this process of assessment. The current authors (WB and AB) developed EMBARK to provide an optimized therapeutic frame for training therapists to support therapeutic benefit in PAP clinical trials. EMBARK is a transdiagnostic, trans-drug PAP model whose structure can be tailored to any PAP intervention that applies a specific psychedelic medicine to a specific clinical indication. It was developed to serve as an adaptable therapeutic framework to be used in all Cybin clinical trials, though we offer it freely to any other entities in the field who would like to adapt it for their own purposes. For example, Anthony Back, MD, collaborated with the current authors and Ladybird Morgan, RN, MSW, to develop an EMBARK-based approach for a clinical trial at the University of Washington School of Medicine that uses psilocybin to treat COVID-related burnout and symptoms of depression in frontline healthcare workers, which included coauthoring an indication-specific EMBARK manual and EMBARK training for study facilitators.

To create EMBARK, the authors surveyed a range of therapeutic approaches that have shown efficacy in conjunction with psychedelic treatment (see Table 1). For the purpose of review, this article divides these approaches into “basic support” and “EBT-inclusive” models, which are distinguished by the lack or presence, respectively, of elements of extrinsic, non-psychedelic evidence-based therapies [EBTs; e.g., Acceptance and Commitment Therapy (ACT), Cognitive Behavioral Therapy (CBT)] that are included in order to provide therapeutic benefit beyond that which is brought about by the administration of a psychedelic medicine in a safe, clinical context.

**TABLE 1:** Existing models of psychedelic-assisted psychotherapy.

	Drug	Indication	Extrinsic EBT(s) or EBT-derived therapeutic approach used
**Basic support models**			
Mithoefer et al. (2017; approach used by Bouso et al., 2008; Mithoefer et al., 2011, 2018; Oehen et al., 2013; Ot’alora et al., 2018; Jardim et al., 2021; Mitchell et al., 2021)	MDMA	Post-traumatic stress disorder	None
Moreno et al. (2006)	Psilocybin	Obsessive compulsive disorder	None
Gasser et al. (2014)	LSD	Anxiety associated with life-threatening illness	None
Carhart-Harris et al. (2016) and Carhart-Harris et al. (2018c)	Psilocybin	Treatment resistant depression	None
Griffiths et al. (2016)	Psilocybin	Cancer anxiety and depression	None
Palhano-Fontes et al. (2019)	Ayahuasca	Treatment resistant depression	None
Davis et al. (2021)	Psilocybin	Major depressive disorder	None
Zeifman et al. (2021)	Ayahuasca	Major depressive disorder	None
**EBT-inclusive models**			
Grob et al. (2011)	Psilocybin	Cancer anxiety and depression	Existential approaches
Johnson et al. (2014)	Psilocybin	Tobacco use disorder	Cognitive Behavioral Therapy (CBT; Quit 4 Life)
Ross et al. (2016)	Psilocybin	Cancer anxiety and depression	Existential approaches, psychodynamic/psychoanalytic, and narrative therapy
Bogenschutz and Forcehimes (2017)	Psilocybin	Alcohol use disorder	Motivational Enhancement and Taking Action (META; based on Motivational Enhancement Therapy) and CBT
Guss et al. (2020)	Psilocybin	Major depressive disorder	Acceptance and Commitment Therapy (ACT)
Anderson et al. (2020)	Psilocybin	AIDS-related demoralization	Brief Supportive Expressive Group Therapy (SEGT)
Monson et al. (2020)	MDMA	Post-traumatic stress disorder	Cognitive Behavioral Conjoint Therapy (CBCT)
Carhart-Harris et al. (2021)	Psilocybin	Depression	Accept Connect Embody (ACE; based on ACT)
COMPASS (2021) and Tai et al. (2021)	Psilocybin	Treatment-resistant depression	Perceptual Control Theory (PCT)

The current article discusses the structure and components of the EMBARK approach and describes how EMBARK functions as a model of PAP and PAP therapist training. The article will first review some of the strengths, limitations, and underdeveloped qualities of prior PAP models in the field (see Table 2) and discuss how the EMBARK model attempts to address them.

**TABLE 2:** Comparative strengths and limitations of basic support and EBT-inclusive models.

	Strengths	Limitations
Basic support models	• Greater participant freedom in meaning-making • Protective of participant autonomy • Less credentialed labor needed • Potential cost savings due to fewer non-medicine session hours	• Missed opportunity for added efficacy • Interventions less operationalized • Staff may be undertrained for challenging clinical and ethical boundary events • Staff not grounded in explicit theory of change may introduce their own • May rely on untested assumption of an “inner healing intelligence”
EBT-inclusive models	• Potential for greater efficacy • Preexisting evidentiary basis • More developed framework for training therapists • Better operationalization of interventions • May require more credentialed staff who can better respond to clinical and ethical challenges • Borrows legitimacy from more established therapies • Provides a sense of competence to PAP-naïve therapists	• Narrowed conceptualization of benefit • Pressure to conform outcomes to predetermined theory of change • Potential invalidation of participant treatment experiences • Possible harm from therapists unprepared for full range of clinical events • Therapists may need to learn new theoretical orientation • Adoption of new orientation may disrupt therapists’ authentic presence • May disregard therapists’ prior expertise • Limits potential to evaluate contributions of various mechanisms of change to efficacy • Unclear if established efficacy of EBTs is carried over into PAP treatment

*Basic support models are those that do not incorporate elements of extrinsic, non-psychedelic evidence-based therapies. EBT-inclusive models are those that do.*

## Assessment of Prior Psychedelic-Assisted Psychotherapy Models

### Common Characteristics of Psychedelic-Assisted Psychotherapy Models

Before contrasting basic support and EBT-inclusive therapy models, it would be helpful to briefly discuss a few notable commonalities between them. As noted earlier, all models of PAP treatment to date have included three phases of treatment. The first phase consists of preparation sessions, which are used to prepare participants to receive the benefits of the medicine. The medicine session(s), referring to the day(s) of drug administration, make up the second phase of treatment. Finally, the third phase, which is often referred to as “integration” (Gorman et al., 2021), consists of a process of reflecting on the medicine session(s) and how it may inspire cognitive and behavioral changes that sustain beyond the end of the treatment.

Another commonality is the universal adoption of an “inner-directed” approach during the medicine session. This refers to a therapeutic stance predicated on “the therapist referencing and encouraging the patient to look into their inner experience for insight and solutions” (Gorman et al., 2021, p. 4). As such, the stance taken by therapists within such an approach is typically limited to interventions that direct the participant’s focus inward. To date, this has been the approach taken by all clinical PAP trials during sessions that involve the administration of a psychedelic medicine (Horton et al., 2021). The differences in therapeutic approach between basic support and EBT-inclusive therapy models, as discussed in this article, are thus found almost exclusively in the preparation and integration phases.

### Strengths and Limitations of Basic Support Models

Several PAP approaches can be meaningfully grouped under the label of basic support models based on their provision of basic, non-psychotherapeutic support to a participant undergoing a course of PAP treatment. The intent underlying this support is non-additive, in that it is not intended to introduce additional therapeutic benefits beyond those thought to be induced by the safe administration of a psychedelic medicine. These approaches rest on the idea that sufficient benefit is obtained by undergoing an inner-directed psychedelic medicine session, framed by supportive preparation and integration sessions, with minimal therapist intervention. In many such approaches, particularly MDMA-assisted approaches (Mithoefer et al., 2017), treatment benefit is thought to depend on an “inner healing intelligence” (Clare, 2018; Gorman et al., 2021) residing in the participant that, when facilitated by the ingestion of a psychedelic medicine and the adoption of an inward attentional focus, will guide the participant toward positive therapeutic outcomes. Preparation sessions are typically limited to informing the participant about events that may occur during the medicine session and encouraging them to develop personal intentions for treatment. Integration sessions typically center on the provision of non-directive, empathic listening and encouragement to the participant as they debrief and make personal meaning of their experience of the medicine session. These approaches do not encourage clinicians to adopt an indication-specific set of interventions or predetermined theory of change from a non-psychedelic EBT.

A noteworthy clinical strength of basic support models is that they allow each participant to make meaning of their inherently unique experience of their medicine session with minimal imposition from any preordained sense of how it should benefit them. These models do not provide clinicians with predetermined frameworks for interpreting treatment benefits and thereby reduce the possibility that they will make poor-fitting interpretations that may disrupt positive treatment outcomes. These models’ interpretive flexibility may also protect participant autonomy as they undergo treatment with drugs that have been known to increase suggestibility (Sjöberg and Hollister, 1965; Carhart-Harris et al., 2015; Timmermann et al., 2020; Dupuis, 2021).

Additionally, these models are likely to be appealing for cost-saving reasons, as the omission of formalized psychotherapeutic interventions means that the provision of PAP treatment may require less credentialed labor. This offers considerable financial advantage given the time-intensive nature of medicine sessions. The non-additive approach of these models also implies that less needs to be accomplished in the non-drug preparation and integration phases, which may lead to fewer treatment hours and further financial benefit.

However, basic support approaches present various significant clinical limitations, several of which are aptly discussed by Sloshower et al. (2020). First, by remaining non-additive and non-specific, these approaches fail to reap the added efficacy that could be gained through the skillful incorporation of evidence-based interventions and theories of change relevant to the indication being treated. Potential synergies between drug effects in the medicine session and indication-specific interventions used in non-drug sessions are left unexplored. Secondly, basic support approaches also present a problem for research rigor insofar as they do not operationalize much of what occurs between the clinicians and the participant during treatment and thereby introduce a significant source of unaddressed variability. Clinicians in prior PAP trials have often applied their prior skills in a way that has not been adequately characterized, which has made it hard to distinguish intervention-independent drug effects from drug effects that synergize with particular therapist interventions.

Additionally, staff provided with minimalist, low-therapy training may be underprepared for challenging clinical situations that can arise during PAP treatment, such as the appearance of trauma or participant boundary-testing. This may contribute to reduced treatment efficacy, increased adverse treatment events, or a greater likelihood of relational boundary transgressions that do significant harm to participants. Finally, the lack of an explicitly proposed, empirically grounded mechanism of therapeutic change in these models may open the door for the inappropriate introduction of therapists’ personal beliefs about how psychedelics heal in a way that may constitute an abuse of their authority (Johnson, 2021) and/or another under-operationalized source of variability in treatment outcomes (Sloshower et al., 2020). These models’ assertion that treatment benefit is brought about by the guidance of an inner healing intelligence, though based on the clinical wisdom of experienced practitioners, has yet to be subjected to empirical scrutiny and may represent another avenue for the introduction of therapists’ personal beliefs about treatment.

### Strengths and Limitations of Evidence-Based Therapies-Inclusive Models

A second cluster of PAP models can be characterized by their treatment of the psychotherapeutic frame in which drug administration occurs as an opportunity to bring additional benefit to participants beyond the medicine session alone by incorporating elements of non-psychedelic EBTs into their clinical approach. Preparation sessions may introduce concepts or tools from an EBT to inform the intentions and quality of inward attention that a participant brings to their medicine session in a way thought to be efficacious for reducing their mental health symptoms. Integration sessions may use a predetermined, EBT-derived framework to sift the elements of a participant’s medicine session experience that best fit a fixed set of desired treatment outcomes. For example, the Yale approach to psilocybin-assisted therapy for depression (Guss et al., 2020; Sloshower et al., 2020) teaches participants the basic elements of psychological flexibility found in ACT as a lens for engaging with their internal experience during their medicine session and for making interpretations and changing their behavior after the medicine session. EBT-inclusive approaches may also include non-drug sessions other than preparation or integration sessions whose format is drawn directly from non-psychedelic manualized treatments, such as Motivational Enhancement Therapy (MET; Bogenschutz and Forcehimes, 2017), to provide additional non-psychedelic psychotherapy to the participant undergoing treatment.

EBT-inclusive models have a variety of strengths in comparison to basic support approaches. For instance, they integrate therapeutic interventions and knowledge from extrinsic, non-psychedelic EBTs that may enhance treatment efficacy beyond what drug administration alone can provide (Thal et al., 2021). Secondly, the incorporation of an extrinsic EBT offers a more developed framework for training therapists and delimiting specified treatment interventions, which enhances the ability to assess treatment fidelity in clinical trials. Thirdly, the employment of credentialed psychotherapists, coupled with the use of more developed therapist training, may also reduce the risk of iatrogenic harm caused by improper clinician responses to challenging clinical events. Fourthly, the adoption of a non-psychedelic EBT incurs the political benefit of borrowing legitimacy from more well-established psychotherapeutic approaches, which is shrewd for a field still in its infancy with an underdeveloped theory of therapeutic action. Finally, the incorporation of language from EBTs and the adoption of an empirically grounded theory of therapeutic change may provide a sense of competence and grounding to therapists, particularly those who are new to PAP, which may enable them to work more effectively with participants. This last point may be an important benefit as the field scales up and recruits large numbers of therapists to meet population-level mental health needs.

Generally, the limitations of EBT-inclusive approaches stem from their introduction of potentially deleterious constraints into how therapeutic benefit is conceptualized in PAP treatment. Therapeutic outcomes in PAP are likely to result from a participant’s engagement with many facets of themselves and their illness during treatment (Kelly et al., 2021; Thal et al., 2021; Miceli McMillan and Jordens, 2022). Medicine sessions can foster a broad range of experiences that inspire participants to make beneficial meaning of their treatment in an equally broad range of ways that include spiritual, existential, emotional, relational, cognitive, or embodied dimensions (Masters and Houston, 1966; Belser et al., 2017; Watts et al., 2017; Malone et al., 2018; Nielson et al., 2018; Michael et al., 2021). The inclusion of a non-psychedelic EBT that was not developed with this breadth in mind may burden the treatment with an overly narrow frame for conceptualizing benefit. At best, this could lead to missed opportunities for therapeutic benefit through the devaluation of phenomena that do not fit its interpretive framework. At worst, it may encourage therapists to pressure participants into conforming their experiences to a fixed set of desired treatment outcomes, which could degrade the therapeutic alliance and invalidate the participant’s personal understanding of a deeply meaningful experience. For example, if a participant’s experience in a medicine session focuses on grieving the loss of a loved one, PAP therapists trained in an ACT-based approach that focuses primarily on increasing psychological flexibility (e.g., Guss et al., 2020; Sloshower et al., 2020) may shift the focus of integration sessions away from the relational or affective dimensions of this experience, which may be most meaningful for the participant, in favor of exploring its metacognitive significance. Harm may then be done to a participant when they lose ownership of the process of making meaning of their experience.

Additionally, training therapists to adopt a constrained understanding of psychedelic phenomena may increase the risk of iatrogenic harm in a way similar to that of basic support models insofar as it underprepares them for the full spectrum of challenging clinical events they may encounter. An EBT-inclusive model firmly rooted in an existing therapeutic approach may also burden study therapists with the challenge of becoming proficient in a potentially novel way of working, while disregarding much of the existing knowledge, skills, and awareness they would otherwise bring to their work. The requirement to work within an unfamiliar therapeutic approach may also be a detriment to therapists’ ability to engage with participants with the type of authentic, abiding presence thought to be important in PAP (Phelps, 2017). Additionally, in a research setting, the adoption of a single theory of change could close off possibilities for noticing and evaluating the contributions of unrelated mechanisms of change outside of the employed therapeutic model. Finally, while much of the appeal of EBT-inclusive models is based on their adoption of EBTs with an established evidentiary basis, it remains unclear whether these EBTs’ proven efficacy is conveyed on the PAP models that attempt to integrate them.

### Lack of Attention to Embodied Phenomena

Most PAP models to date have paid little attention to the role of embodied events in therapeutic outcomes, despite their prevalence in participants’ accounts of their medicine experiences (Belser et al., 2017; Watts et al., 2017). This inattention may reflect the ongoing effort to characterize PAP within the institutional bounds of Western psychiatry and psychology, which attend more exclusively to cognitive and neural phenomena. As such, beneficial outcomes of PAP treatment have been conceptualized in neuropsychological terms that omit participants’ experiences of embodiment (Kelly et al., 2021; Thal et al., 2021), such as the “reset” of a maladaptive neural status quo (Carhart-Harris et al., 2017; Carhart-Harris and Friston, 2019), increased psychological flexibility (Watts and Luoma, 2020; Agin-Liebes et al., 2022), cognitive reappraisal (Agin-Liebes et al., 2022), altered neural responses to affect (Barrett et al., 2020), or increased neuronal and mental plasticity (Kočárová et al., 2021). This centering of the brain-mind may seem unremarkable, given the prevalence of neuropsychological explanations offered by most psychotherapies. But its appropriateness is less clear when applied to the more body-inclusive experiences elicited by psychedelic medicines.

Several qualitative studies of PAP participant experiences (Belser et al., 2017; Watts et al., 2017; Bogenschutz et al., 2018) include accounts of unprompted somatic phenomena that arose in PAP medicine sessions and were experienced by participants as important to their treatment. They included interoceptive experiences of locating undesirable psychic content (e.g., grief, shame, resentment, anger) or physical illness (e.g., cancer, sequelae of problem drinking) in their lived experience of their bodies, as well as “purgative,” “purifying,” (Watts et al., 2017, p. 550) or “washing” (Belser et al., 2017, p. 370) experiences that diminished the perceived personal impact of these maladies, sometimes by way of vomiting or spitting (Bogenschutz et al., 2018). These participants’ experiences of discharging unwanted material resonate with some indigenous frameworks for the use of psychedelic medicines that consider various forms of embodied “purging” to be instrumental in healing (Fotiou and Gearin, 2019). They also bear a similarity to phenomena suggested as potentially therapeutic by a range of understudied somatic psychotherapy approaches that have grown outside the walls of academic research, such as Somatic Experiencing (Levine, 1997) and the Trauma Resiliency Model (Grabbe and Miller-Karas, 2017). These approaches suggest that life events that adversely impact psychological functioning, such as traumas, also leave a harmful memory trace in one’s body that can be “discharged” through somatic events like sobbing, involuntary shaking, or trembling (Van der Kolk, 1998). The omission of these and other somatic phenomena from most prior PAP models may thus reflect cultural bias and a carrying over of the institutional blind spots of academic Western psychiatry and psychology.

To date, PAP clinical trials have considered the mind to be the locus of therapeutic outcomes while the body is the site of unwanted adverse treatment events, such as nausea, paresthesia, or pain with no organic cause. We suggest that earlier PAP models (with few exceptions; see Mithoefer et al. (2017), Gorman et al. (2021)) may have ignored the therapeutic relevance of these and other embodied phenomena to the detriment of treatment efficacy, and we would argue that a considered incorporation of practices for supporting and responding to somatic events skillfully and ethically may enhance treatment outcomes.

### Lack of Attention to Relational Elements of Treatment

PAP models to date have primarily framed treatment benefit from within what Barnes and Briggs (2021) have called a “one-person psychology,” in that they view therapeutic change as emanating from an internal, subjective process that occurs during a medicine session. In most PAP clinical trials to date, particularly those with classic psychedelics, participants have been invited to don an eye mask and headphones for the duration of the medicine session and “go inward” to have an inner-directed experience with limited intervention from clinicians (Horton et al., 2021). However, some participants have still opted to engage interpersonally with the clinicians from within the altered relational dynamics of a medicine session. These dynamics are likely to differ significantly from those found in talk therapy and may include heightened participant suggestibility, vulnerability, and sensitivity; alterations in the meanings and expectations that the participant ascribes to the relationship; amplification of the participant’s attachment style or other habitual relational behavior; and increased participant-led boundary testing (Sjöberg and Hollister, 1965; Passie, 2012; Carhart-Harris et al., 2015; Taylor, 2017; Dupuis, 2021). Additionally, even if a participant remains inward for the full duration of a medicine session, they may experience increased feelings of social connectedness and emotional empathy (Pokorny et al., 2017; Carhart-Harris et al., 2018b) that will subsequently impact their relationships post-medicine, including those with the clinicians during the subsequent integration phase.

Prior PAP approaches to working with classic psychedelics have provided clinicians with little guidance on how to engage with these altered relational dynamics in a way that contributes to therapeutic benefit. For basic support approaches, this is likely due to the general lack of hands-on guidance they offer therapists. EBT-inclusive models have likely omitted this relational focus due to their reliance on extrinsic EBTs that lack a relational focus, such as third-wave behavioral approaches (Walsh and Thiessen, 2018). However, Mithoefer et al. (2017) have suggested that the altered therapist-participant dynamics found in MDMA-assisted treatment may provide unique opportunities for relational repatterning work between participants and clinicians that could lead to durable improvements in social and psychological functioning. Elsewhere, it has been suggested (Barnes and Briggs, 2021; Barnes, 2022) that similar opportunities might be present in treatment with classic psychedelics as well. In support of this notion, a recent survey study found that individuals who attended group psychedelic ceremonies and felt they had a positive relationship with the ceremony facilitators self-reported greater improvements in wellbeing than those who did not (Kettner et al., 2021). Additionally, Murphy et al. (2022) recently found that the strength of the therapeutic relationship in the preparation phase of a course of psilocybin-assisted treatment for depression predicted greater emotional-breakthrough and mystical-type experience in the medicine session, which in turn led to greater reductions in depressive symptoms. The lack of attention paid by most current PAP models to relational aspects of treatment may represent another under-characterized avenue of therapeutic benefit in PAP and a missed opportunity for therapist training.

### Insufficient Focus on Ethics

The altered relational dynamics found in PAP treatment may also present novel relational ethical challenges that fall outside of the scope of clinicians’ prior professional training in ethics. These challenges often stem from the effects of the psychedelic medicine on a participant’s subjectivity, which include increased suggestibility, disruption of interpersonal boundaries, heightened transference, attempts to reenact of traumatic early life dynamics, and increased tendency to ascribe great wisdom or power to therapists (Harlow, 2013, as cited in Passie (2018), p. 12; Taylor (2017); Northrup, 2019). Failure to navigate these challenges may lead to a variety of transgressions that could harm participants, such as therapist sexual abuse, other forms of harmful touch, confusion of the therapeutic frame, or the inappropriate imposition of the therapist’s ideas. Several high-profile cases of sexual abuse in research (Goldhill, 2020) and underground (Hall, 2021) settings corroborate these concerns of relational harm, as do published warnings from respected PAP researchers about other sources of relational harm risk specific to PAP (Anderson et al., 2020; Johnson, 2021).

There remains an unmet need for a more commensurate response to this added risk of harm in PAP treatment. Responses so far have included the development of psychedelic-specific codes of ethics for practitioners (MAPS, 2019), attentiveness to personal risk factors in trial therapist supervision (Tai et al., 2021), and reminders placed in therapist training manuals to maintain healthy boundaries (Mithoefer et al., 2017; Guss et al., 2020). However, therapist training in PAP clinical trials has yet to devote adequate attention to ethical concerns despite their central importance in the provision of safe, efficacious care to participants. The field has struggled to rise to the full breadth of the ethical responsibility that PAP treatment warrants, which includes not only the prevention of boundary transgressions, but attentiveness to other areas where ethical competence is needed, such as attending to cultural considerations (Fogg et al., 2021), ensuring that structural injustices are not replicated in treatment settings (Michaels et al., 2018; George et al., 2020), and providing care that is mindful of the understudied risks to participant wellbeing posed by psychedelic medicines, particularly in the presence of trauma (Haridy, 2021; Love, 2022). Taken together, these concerns represent areas for ethical growth to which future PAP models should attend.

## How the EMBARK Approach Responds to These Challenges

The authors developed the EMBARK model of PAP and PAP training as a response to these concerns. The following section describes how the EMBARK model addresses the limitations of the existing basic support and EBT-inclusive models discussed so far.

### Conceptualizing Therapeutic Change

The EMBARK approach conceptualizes change in PAP treatment in a way that avoids both the agnosticism of basic support models and the constrictive overdetermination of EBT-inclusive models. Its essential structure is made up of six clinical domains, or parallel conceptual avenues by which benefits may arise, which reflect the model’s openness and responsiveness to the plurality of ways in which participants may benefit from PAP. They form an acronym that gives the approach its name: Existential-spiritual, Mindfulness, Body-aware, Affective-cognitive, Relational, and Keeping momentum. Each domain represents a conceptual through line in PAP treatment that organizes a cluster of possible in-session events in a way that facilitates working with these events therapeutically. This multimodal organization allows the model to have a structured sense of how to prepare therapists to support participants without losing sight of the diversity of paths that each unique participant’s course of treatment could take. Each individual participant’s treatment experience will likely only occur within one or a few of these domains, and the selection process is guided by the natural unfolding of the participant’s therapeutic process. The inherent unpredictability of this unfolding necessitates that EMBARK therapists be trained to competence in all six domains, and taught the skills to flexibly employ the relevant domains, prior to the start of treatment. These six domains will be discussed in more detail later.

### Incorporating Extrinsic Evidence-Based Therapies

EMBARK was designed to be capable of curating helpful elements from several non-psychedelic EBTs and other therapeutic approaches without wedding itself to one. This allows it to reap the benefits of extrinsic EBTs—helpful conceptual frameworks, efficacious treatment goals—without sacrificing the model’s conceptual multiplicity by adopting a single, constrictive theory of change. EMBARK’s six-domain structure facilitates the infusion of elements from multiple indication-specific EBTs that support therapeutic outcomes in each domain. For example, an EMBARK approach to treating alcohol use disorder may incorporate elements of Mindfulness-Based Relapse Prevention (MBRP; Bowen et al., 2009) in its “Mindfulness” domain, Cognitive-Behavioral Therapies (CBTs) in its “Affective-cognitive” domain, and Motivational Interviewing (MI; Riper et al., 2014) in its “Keeping momentum” domain. An EMBARK approach to another indication would likely incorporate elements of different EBTs shown to be efficacious in treating that indication. In any case, EMBARK’s coherent overarching structure enables all incorporated techniques to coexist in a clear, meaningful, and synergistic way. As a participant’s unique course of treatment begins to favor one domain over another, EMBARK therapists can draw more heavily from the extrinsic EBTs associated with that domain and guide the treatment toward outcomes that are in line with that domain’s proposed mechanism of therapeutic change.

### Delimiting Interventions

As noted earlier, basic support approaches give therapists little sense of what interventions they should or should not use, while EBT-inclusive approaches can be prescriptive about this in a way that often asks therapists to work outside of their expertise. To avoid these issues, EMBARK therapists are instead provided with a set of treatment tasks for each phase of treatment (e.g., “help the participant understand the importance of approaching challenging feelings and beliefs in the medicine session”) that can be completed by way of a wide range of interventions. In carrying out these tasks, therapists are encouraged to use whichever interventions and modalities they are most comfortable and experienced with, as long as they abide by a set of general guidelines laid out in the treatment manual (e.g., “be respectful of the participant’s hesitance about approaching confronting personal material”). This way of providing flexible structure eases the burden on therapists to become proficient in novel clinical approaches and allows them to draw on their existing skill sets. Having clearly defined tasks and guidelines also facilitates the operationalization of what needs to be accomplished in sessions for the purposes of standardization.

### Determining Contributing Factors in Treatment Outcomes

A clinical trial that uses an EBT-inclusive model runs the risk of improperly characterizing the factors that contribute to efficacy. For instance, successful treatment outcomes in an MET-based PAP trial treating substance abuse may be conceptualized in the language of reduced ambivalence and increased motivation for behavioral change, when participants’ success in abstaining may be due more to the alleviation of mental pain by way of somatic trauma processing or enhanced resilience conferred by spiritual factors. EMBARK’s pluralistic approach to mechanisms of change, reflected in its six domains, gives clinicians and researchers a framework that could support less biased, more exploratory inquiries into what treatment events support benefit, while still providing sufficient structure for proposing specific, empirically informed hypotheses. As researchers begin to address the question of how PAP has its therapeutic effects, the EMBARK model presents a compelling way to operationalize, examine, and disentangle the impact of a broad spectrum of treatment events.

## EMBARK’s Structure

The following section provides an explanation of EMBARK’s foundational structure: its six clinical domains, four care cornerstones, and three phases of treatment. Several of the domains reflect EMBARK’s incorporation of the neglected elements of PAP treatment discussed above, and the care cornerstones demonstrate the model’s centering of ethical concerns in its approach to therapist training. The final sub-section on the three phases of treatment provides an explanation of how the other elements of EMBARK’s structure come together into a unified treatment approach.

### EMBARK’s Six Clinical Domains

Each EMBARK domain refers to a set of related treatment events that can bring about therapeutic benefit if worked with in a way that is responsive to their unique requirements. For each domain, an EMBARK approach to a specific indication includes (1) one or more proposed mechanisms of therapeutic change, (2) specific therapist tasks and guidelines for interventions that support these mechanisms, and (3) indication-specific treatment goals that follow meaningfully from phenomena in the domain. Therapists are tasked with determining when one or more of these domains become salient in a participant’s treatment and providing them with the support required to obtain benefit within this domain. In this section, each domain will be described generally, and an example of how therapeutic interventions in this domain might look will be drawn from the EMBARK manual for the study of psilocybin in the treatment of major depressive disorder (MDD).

#### Existential-Spiritual

Psychedelic medicines are well known to catalyze profound encounters with mystical or spiritual content (Griffiths et al., 2006; Breeksema et al., 2020; Podrebarac et al., 2021) and existential concerns, such as mortality (Swift et al., 2017), alienation (Watts et al., 2017), or questions of life meaning (Ross et al., 2016, 2021; Belser et al., 2017). This is perhaps not surprising, given the historical and current use of psychedelic plants and fungi in the religious practices of many cultures worldwide (Schultes et al., 2001). Several PAP clinical trials have found that participants often report profound experiences of an existential or spiritual nature (Johnson et al., 2014; Bogenschutz et al., 2015; Belser et al., 2017; Podrebarac et al., 2021) that may hold enduring significance for them (Griffiths et al., 2006). The potency of participants’ mystical experiences has been found to correlate with a range of treatment benefits, including reductions in symptoms of depression (Davis et al., 2021) and treatment-resistant depression (Roseman et al., 2018), increased motivation to stop problematic cocaine use (Dakwar et al., 2014), decreases in cancer-related depression and anxiety (Griffiths et al., 2016; Ross et al., 2016), greater success in nicotine cessation (Johnson et al., 2014), and other positive changes in psychological functioning (Griffiths et al., 2018). Despite calls for further elucidation of specific therapeutic mechanisms in this domain (Roseman et al., 2018; Breeksema and van Elk, 2021; Jylkkä, 2021; Sanders and Zijlmans, 2021), existential and spiritual elements of PAP warrant recognition as potential sources of treatment benefit (Johnson et al., 2019).

The current state of our knowledge suggests that anti-depressive outcomes may be facilitated simply by having a mystical experience during a medicine session (Roseman et al., 2018; Davis et al., 2021). However, providing support for a participant’s post-medicine spiritual self-development may contribute additional benefit, as suggested by prior PAP research (Griffiths et al., 2018; Lafrance et al., 2021) and non-psychedelic findings of negative correlations between spirituality and depressive symptoms (Koenig, 2009; Bonelli et al., 2012). The role of EMBARK therapists treating MDD by way of this domain is thus to create the conditions for mystical or spiritual phenomena to potentially arise and to support participants in using them as an impetus for spiritual growth. In the preparation phase, therapists assess for any intrinsic motivation a participant may have to bring existential-spiritual elements into their treatment and work with them in developing this motivation into their intentions for the medicine session. For the medicine session, therapists prepare the physical treatment space in a way that demonstrates respect for the subjective sense of sacredness that may arise for the participant and open the session with a brief, collaboratively designed ritual. If phenomena in this domain arise during the medicine session, therapists are invited to use supportive psychotherapy or evidence-based interventions of their preference during the integration phase to explore the participant’s experience, support them in developing a sense of what actions it could motivate, and co-create plans for life changes and/or further spiritual self-development. Examples of approaches with evidentiary bases to use in this process include meaning-oriented psychotherapies (Vos et al., 2015), Logotherapy (Thir and Batthyány, 2016), or Spiritual Guidance (Miller et al., 2008), which is derived from the evidence-based approach of MI (Riper et al., 2014), despite not being an EBT itself. Throughout all phases, therapists are taught to attend to their own biases and beliefs in order to avoid imposing their own understanding or interpretation on a participant’s experience in this domain.

#### Mindfulness

This domain refers to treatment events that result in the participant becoming more capable of recognizing symptomatic internal states and responding to them with a greater capacity for self-compassion and self-regulation. Mindfulness in EMBARK has significant conceptual overlap with the notion of “psychological flexibility” that the ACE Model (Watts and Luoma, 2020) derives from ACT. In previous PAP trials, participants have often experienced various forms of disruption of their habitual self (Belser et al., 2017; Roseman et al., 2018), a sense of “mental freedom” (Watts and Luoma, 2020, p. 95), or an increased feeling of sovereignty in how one relates to the workings of their own mind (Bogenschutz et al., 2018). Overall, the “M” domain represents a place in EMBARK for preparing the participant to attend to their thoughts and feelings with compassion during a medicine session and for working with increases in psychological flexibility and other metacognitive shifts during integration.

In the treatment of MDD, mindfulness has been found helpful in disrupting ruminative thought patterns (Watkins, 2016), enabling more cognitive flexibility (Kohtala et al., 2018; Shapero et al., 2018), and fostering a more compassionate stance toward oneself (Segal et al., 2012). To support these outcomes, EMBARK therapists begin a course of PAP treatment for MDD by teaching basic mindfulness skills to the participant. This teaching is only meant to ensure that the participant knows how to attend to their internal experience during the medicine session, which is when a more enduringly increased capacity for mindfulness may arise. During the integration phase, therapists help to anchor whatever aspect of mindfulness arose for the participant through the use of indication-specific mindfulness practices. Therapists may also help the participant integrate a new feeling of self-compassion or support them in using the momentary abatement of ruminative thoughts as an opportunity to develop mindfulness-based skills that may prevent a recurrence. Therapists can use their own mindfulness-based interventions within the bounds of the guidelines provided, or they can use suggested interventions drawn from Rumination-Focused CBT (RF-CBT; Watkins, 2016) and Mindfulness-Based Cognitive Therapy (MBCT; Segal et al., 2012) included in the treatment manual. At all times, therapists are taught to work with the participant in this domain within a trauma-informed approach to mindfulness and to avoid imposing one’s own beliefs or biases onto the participant’s experience.

#### Body Aware

As discussed earlier, PAP participants have reported that embodied phenomena are a notable part of their experience of a medicine session. There are few EBTs that provide support in conceptualizing or responding to these phenomena, though innovative somatic psychotherapy approaches have offered suggestions (Levine, 1997; Grabbe and Miller-Karas, 2017). EMBARK therapists are prepared to respond to embodied treatment events using the most widely accepted elements of these novel somatic approaches, such as “pendulation,” or the process of helping a participant alternate between active, embodied engagement with trauma material and self-soothing (Grabbe and Miller-Karas, 2017). EMBARK also encourages integration of somatic elements from more established EBTs, such as somatic awareness training exercises and self-regulation skills from MBRP or Dialectical Behavioral Therapy (DBT; Linehan, 2014) to support therapeutic outcomes in this domain.

Importantly, the techniques used by EMBARK therapists in this domain do not require them to work with the body in an intensive, hands-on way and are thus not prohibitively far beyond their standard psychotherapeutic training. In preparation, therapists train the participant in basic somatic awareness so that they can attend to and thereby facilitate pro-therapeutic bodily phenomena during the medicine session. Once the medicine is administered, the therapists’ role is to guide the participant back toward an awareness of their body when clinically indicated and help them remain within their zone of optimal arousal through the use of the self-soothing interventions discussed earlier or therapist-participant touch-based interventions.

While some have asserted the importance of therapist-participant touch in PAP (Mithoefer et al., 2017; McLane et al., 2021) based on historical assertions by authorities in the field (e.g., Martin, 1957; Eisner, 1967), others have noted that there remains a lack of knowledge or consensus around the efficacy, safety, or necessity of therapist-participant touch in PAP (Devenot et al., 2022). EMBARK therapists are thus instructed to prioritize non-touch interventions and limit their touch-based interventions to basic supportive touch that minimizes points of contact between parties, like handholding or placing a hand on a participant’s shoulder to convey support or offer grounding. More intensive forms of touch (e.g., full-body embraces) are omitted until further research establishes them as safe, effective interventions. Therapists also rigorously assess for the participant’s consent to touch-based interventions during preparation and proactively give participants a chance to reject any form of touch during preparation, immediately before the provision of touch in the medicine session, and at any time during the touch, so as not to move beyond the participant’s espoused level of comfort.

Since most interventions in this domain involve supporting the participant in mindfully attending to their body, the “B” domain shares some practical overlap with the “M” domain. The primary distinction between the Body aware and Mindfulness domains is found less in their associated interventions than in participants’ subjective experiences of how benefits arise and the integration goals that best support these benefits. Body aware treatment goals follow from medicine session events that a participant locates in their body (e.g., “I saw the [pain in my chest] […] then I felt so much lighter, like something had been released,” Watts and Luoma, 2020, p. 95) and involve integration practices that work with shifts in somatic awareness. Treatment goals in the Mindfulness domain involve benefits that adhere more closely to the core concepts of psychological flexibility and other metacognitive shifts (“I got a wider perspective […] that there’s a lot more going on than just the minor things that were going on in my head,” Watts and Luoma, 2020, p. 96) and are sustained with practices that build upon a participant’s revised relationship with the contents of their mind.

In the treatment of MDD, somatic phenomena are hypothesized to contribute to therapeutic outcomes in two ways. Depressive symptoms are notable for their disturbance of embodiment (e.g., fatigue or energy loss, disruptions of sleep and appetite, weight gain or loss). It has been suggested (Ratcliffe, 2015; Doerr-Zegers et al., 2017; Rønberg, 2019) that disruptions of one’s lived experience of their body are the most fundamental and cross-culturally consistent MDD phenomena. As such, the experiences of enlivenment and sensory embodiment that some PAP participants describe (Watts et al., 2017) may form the basis for a relationship with one’s body that opposes the recurrence of depressive symptoms. If such an experience arises for a participant, therapists may work with them to build and strengthen this new relationship.

Additionally, it is likely that treating MDD will often involve working with trauma. The comorbidity and symptomology of MDD and trauma has been posited as evidence that many cases of MDD can be meaningfully thought of as a subtype of PTSD characterized by an internalizing response to trauma (Flory and Yehuda, 2015). Although classic psychedelics have not yet been used to address trauma in a completed clinical trial, it has been suggested (Bird et al., 2021) that the disinhibiting effect of psilocybin on frontal-limbic neural circuits of emotional regulation are similar to those of MDMA, which has more established efficacy in treating trauma (Bahji et al., 2020; Illingworth et al., 2021; Smith et al., 2022). Classic psychedelics may thus have facility in trauma treatment by way of similar disinhibitory mechanisms. It is likely that PAP treatment of MDD with a classic psychedelic will sometimes facilitate the appearance of trauma symptoms, possibly in the form of the somatic phenomena described earlier (nausea, shaking, etc.). When this occurs, a skillful therapeutic response may facilitate lasting resolution.

#### Affective-Cognitive

During a medicine session, some PAP participants experience dramatic shifts in their emotions and cognition. Participants have often reported that they experience a degree of emotionality that is ordinarily unavailable to them, including feelings of bliss, love, despair, fear, and grief, often in the context of a healing catharsis (Belser et al., 2017; Watts et al., 2017; Breeksema et al., 2020). They may also find themselves confronting maladaptive self-beliefs with a directness they would normally avoid (Bogenschutz et al., 2018). Engaging non-defensively with these emotions and beliefs during a medicine session has become a widely accepted practice among PAP models. This practice mirrors that found in several non-psychedelic EBTs, such as ACT’s emphasis on acceptance (Hayes et al., 2012) and Emotion-Focused Therapy’s (EFT; Greenberg and Watson, 2006) emphasis on entering into maladaptive states in service of transformation. This domain serves as a space within EMBARK for incorporating approach-oriented practices for working with emotions and self-beliefs in way that best addresses the indication being studied.

For example, the EMBARK approach to MDD in this domain begins with the notion that many depressed individuals have developed a habitual response to challenging feelings that entails dimming their awareness of them through a kind of automatic, unconscious avoidance and characteristic depressive experience of feeling numb and withdrawn (Tull et al., 2004). Participants in PAP medicine sessions have often experienced a greater facility in reconnecting with this avoided material and have implicated these experiences in their positive treatment outcomes (Gasser et al., 2015; Watts et al., 2017; Bogenschutz et al., 2018; Watts and Luoma, 2020). In the preparation phase of MDD treatment, EMBARK therapists are tasked with helping participants understand the importance of adopting an approach orientation during the medicine session. They also help the participants develop one or more self-soothing techniques. During the medicine session, the therapists’ role is to remind the participant to welcome challenging content if needed and to help them utilize the previously learned self-soothing techniques when necessary. To integrate these experiences, EMBARK therapists help participants use experiences of approaching challenging material as the basis for updating maladaptive core beliefs about oneself or the world and cultivating an enduring attitude of greater acceptance in their emotional life. All of these interventions are taught and employed in a way that is respectful of participant autonomy, the principles of trauma-informed care, and cultural differences in relating to and expressing one’s emotions.

#### Relational

As noted earlier, practitioner-participant interactions are often significant events in medicine sessions that have yet to be adequately characterized by other PAP models. These interactions may be home to amplified versions of dynamics that are typically found in any relational form of psychotherapy, including transferences, projection, or reenactments of traumatizing dynamics or events. As in talk therapy, the appearance of these phenomena represents an opportunity for clinical gain if handled skillfully or rupture and harm if handled poorly. EMBARK therapists are thus trained to work ethically and efficaciously in this domain through the didactic and experiential processes discussed later under the Ethically rigorous care cornerstone.

In the treatment of MDD, these relational events are framed as potential moments of relational repatterning that may reduce depressive symptoms. A core element of depression is social isolation, both actual and felt (Meltzer et al., 2013; Morrison and Smith, 2018). This isolation may derive from patterns or beliefs learned in early life relationships, such as a sense that one is unacceptable, deserves to be alone, or may lose love if they express themselves freely in the presence of another person. The altered relational dynamics of a PAP medicine session may provide opportunities for relational repatterning that supplants these maladaptive beliefs. EMBARK therapists are also prepared to support relational benefits that may arise for the participant outside of their interactions with the therapists, such as an internally felt sense of social connectedness or emotional empathy (Pokorny et al., 2017; Carhart-Harris et al., 2018b) or an autobiographical review process that examines past and present relationships in the participant’s life (Belser et al., 2017; Watts et al., 2017). If these shifts in social cognition occur and are skillfully worked with in the integration phase, a depressed participant can be helped to use them as the grounds for a less isolative social life.

#### Keeping Momentum

A course of PAP treatment is brief, and its most enduring benefits have been conceptualized as those that continue to unfold well beyond the final session (Mithoefer et al., 2017; Dölen and Briggs, 2021). PAP participants often emerge from a medicine session with a sharp uptick in the sense of motivation, self-efficacy, and commitment to making pro-therapeutic changes to their behavior or life context (Bogenschutz et al., 2018; Griffiths et al., 2018; Nielson et al., 2018). Some may also develop a clarified sense of their deeply held values, which may form the basis for beneficial post-treatment actions (Watts and Luoma, 2020). At the neural level, it has been suggested that the plasticity brought about by many psychedelic medicines may signal the reopening of a social reward critical learning period for weeks after administration, suggesting that pro-therapeutic changes may take root during a time period that extends beyond the end of what is typically considered integration (Nardou et al., 2019; Dölen, 2021; Dölen and Briggs, 2021; Lepow et al., 2021). The EMBARK approach recognizes this unique opportunity. Therapists are trained to support a participant’s movement from the setting of intentions to the planning of concrete actions by attending to importance of post-treatment changes throughout all stages of PAP treatment.

For MDD or any other indication under study, these changes are expected to look very different for each participant. EMBARK therapists are trained to apply a broad lens to what helpful post-treatment change might look like. For some participants, the most supportive change may be at the level of personal behaviors, such as problem drinking or procrastination. For others, change may be warranted in their personal contexts, such as relationships or work environments. Some participants may find additional benefit in taking aim at collective concerns that have a bearing on their life, such as structural racism or exploitative work conditions, through participation in collective organizing. The EMBARK approach recognizes the potential of both individual and collective forms of change in the service of enhancing a participant’s psychological, spiritual, and social wellbeing.

### EMBARK’s Four Care Cornerstones

The EMBARK approach rests upon four pillars of ethical care. They represent the model’s commitment to the notion that efficacious treatment is inextricably linked with ethical treatment. These pillars are woven into all levels of any EMBARK approach to a specific indication, from conceptualization to treatment. Subject matter experts were invited to teach an EMBARK training module on each cornerstone (see below), and key elements from their teachings have been written into EMBARK manuals.

#### Trauma-Informed Care

This cornerstone reflects a recognition of the prevalence of trauma likely to be found in PAP clinical trial participants. Moreover, it marks the need for greater attentiveness to the unique and sensitive ways that trauma may manifest in PAP medicine sessions and the potential for retraumatization that may occur in the absence of adequate training. Therapists trained in the EMBARK approach are provided with psychedelic-specific training in how to identify and respond to trauma when it arises, ways to avoid re-traumatization, and strategies for maintaining their own wellbeing in service of sustainably supporting participants.

Marcela Ot’alora G., MA, LPC, contributed her approach to trauma-informed care to the EMBARK training program. Her training materials applied the Substance Abuse and Mental Health Services Administration’s (2014) SAMHSA six key principles of trauma-informed care (safety; trustworthiness and transparency; peer support; collaboration and mutuality; empowerment, voice, and choice; cultural, historical, and gender issues) to PAP treatment, along with the additional principles of choice and autonomy. These principles were discussed in conjunction with important practical considerations and attention to ways in which therapists may unwittingly cause harm to participants who carry trauma. EMBARK manuals emphasize these principles and considerations throughout treatment, with particular attention paid to ensuring that any exercises that invite the participant to focus inwards (e.g., mindfulness, somatic awareness) are conducted in a way that is sensitive to the needs of those with trauma.

#### Culturally Competent Care

The field of mental health has reached a consensus that the ability to provide care to those who differ from their therapist in terms of race, culture, gender, sexual orientation, or class is an essential part of delivering effective treatment (American Psychological Association, 2017). However, training therapists to consider these factors when treating those who are culturally different has often lagged behind this realization. This has been particularly true in clinical PAP trials, which have received much scrutiny for not recognizing the importance of cultural considerations (Herzberg and Butler, 2019; George et al., 2020; Williams et al., 2020; Fogg et al., 2021). While an appropriate response to these concerns would entail structural and cultural changes in the field of PAP research that go beyond therapist training, EMBARK training was designed to minimize iatrogenic harm by helping therapists to engage in culturally humble and attuned ways that avoid replicating oppressive dynamics in the therapist-participant relationship.

NiCole T. Buchanan, Ph.D., has led the training module on this cornerstone. She has provided materials for therapists on a variety of areas pertaining to cultural competence in clinical work: privilege, power, implicit bias, awareness of one’s social position, concerns around language and non-verbal communication, cultural influences on the expression of emotions and disclosure of struggles, norms around touch and relationships, use of the DSM-V Cultural Formulation Interview (American Psychiatric Association, 2013), the adoption of an anti-oppressive advocacy approach to the role of therapist (Ali and Lees, 2013), and more. Psychedelic-specific topics that she addressed in this module include the influence of the War on Drugs on People of Color seeking psychedelic treatment, responding competently to intergenerational and collective content that arises in medicine sessions (e.g., cultural grief), and considering cultural appropriation in music selection. Attentiveness to these topics have been incorporated into EMBARK manuals’ approaches to PAP treatment.

#### Ethically Rigorous Care

As noted earlier, PAP treatment presents unique ethical challenges that, if mishandled, could lead to boundary transgressions that do considerable harm to participants. EMBARK responds to this heightened potential for relational harm by interweaving ethical considerations and training into many aspects of its approach, including the guidelines it offers for therapist interventions. EMBARK therapists are also supported in their own ethical self-reflection and growth through ongoing supervision, participation in peer consultation groups, and other practices that aim to enhance their ethicality, as therapists’ own self-reflection and growth is central to their capacity to engage with participants in ethically grounded ways. The model also recognizes the importance of organization-wide commitments to preventing and responding to boundary transgressions with integrity and encourages those who employ EMBARK to adopt such commitments.

Kylea Taylor, MS, LMFT, has taught this module in the EMBARK training. She presented therapists with techniques for attending to how their personal material may be activated by the unique demands of PAP work. Her didactics touched on considerations around touch, multiple relationship, power differentials, suggestibility, and other psychedelic-relevant topics not adequately covered by therapists’ standard ethical training. Attentiveness to these elements is also written into EMBARK manuals’ instructions on setting relational boundaries, being mindful of and proactively discussing relational dynamics with participants, assessing consent for touch, and other ethical practices. Suggested practices for continued ethical development are discussed in an appendix found in all manuals as well.

#### Collective Care

The underlying causes of the struggles and symptoms that bring PAP participants into treatment are not always entirely locatable in the personal and biological conditions of their lives. The fields of medicine and psychology have become increasingly attentive to the fact that the micro-level conditions they treat are heavily influenced by the macro-level or structural conditions of the society in which participants live (Ali and Sichel, 2014). These societal factors include discrimination built into governing institutions (e.g., legal systems, policing), inequities in access to resources (e.g., housing, medical care, work, social services), structural deficits in community cohesion and mutual support, and systemic inattentiveness to those with increased needs (e.g., those with disabilities, the elderly). EMBARK therapists are trained to attend to structural factors in their work and are offered recommendations on how they can broaden their sense of their role and become more holistic advocates for the participants they have committed to serve. Additionally, the model recognizes that much of the onus for collective care falls upon the organizations that employ it and thus encourages any groups who use EMBARK to commit resources to addressing structural concerns relevant to the populations they serve.

Florie St. Aime, LCSW, led the EMBARK training in this cornerstone. She facilitated an exploration of psychotherapy and PAP’s situatedness within deeply engrained systems of domination, anthropocentrism, and historical inequities, with a focus on the implications this has for how suffering is treated in PAP clinical trials. Her presentation also focused on historical and ancestral dimensions that may arise in PAP and what it would mean to respond to them with more collective notions of care. In the EMBARK manuals, this cornerstone shows up most notably in the integration phase, which holds that pro-therapeutic, post-treatment change at the personal level can be most supportive to participants when it occurs in conjunction with changes to the broader structural context of their lives.

### EMBARK’s Three Phases of Treatment

The EMBARK model adheres to the three-phase PAP treatment design that has been used in all clinical trials published to date (Horton et al., 2021). It consists of non-drug preparation sessions prior to administration, medicine sessions in which the psychedelic medicine is administered, and non-drug integration sessions after the date of administration.

**FIGURE 1:**
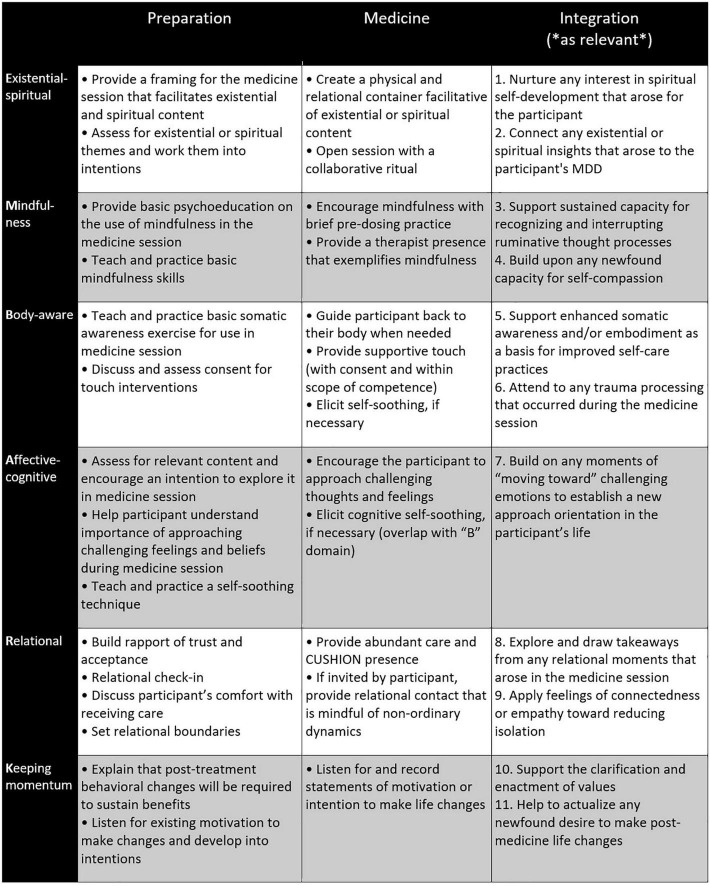
Domain-specific therapist tasks across three treatment phases of EMBARK approach to MDD. Integration aims are numbered to correspond to the integration aim guidance checklist (Figure 3).

**FIGURE 2:**
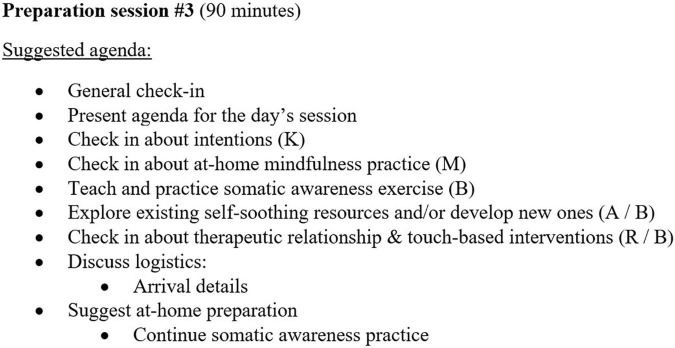
Example agenda for preparatory session #3, final preparation session before medicine session in EMBARK approach to MDD. Letters in parentheses indicate the domain supported by each task.

**FIGURE 3:**
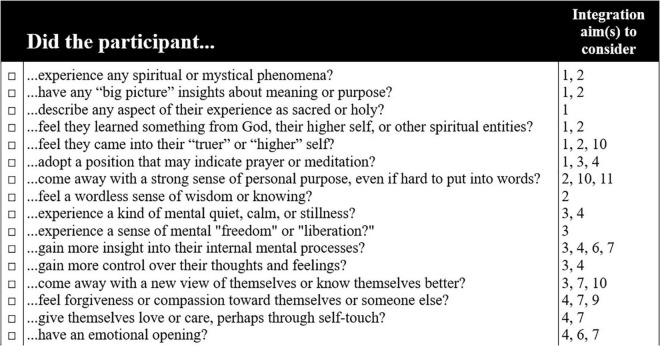
Portion of integration aim guidance checklist provided in EMBARK manual for MDD treatment. Numbers in the “Integration aim(s) to consider” column refer to suggested integration aims detailed in the rightmost column of Figure 1.

For the sessions within each phase, therapists are given a set of general tasks, as well as domain-specific tasks for each of the six domains (see Figure 1 for examples of domain-specific tasks in all three phases). For the preparation and medicine phases, these two sets of tasks are woven together into suggested agendas for each session, which therapists can apply with flexibility and responsiveness to the needs of a specific participant (see Figure 2 for an example agenda). In the integration phase, therapists and participants collaboratively choose which integration goals they will pursue based on their pertinence to what arose in the participant’s medicine session (see Figure 1 for examples of integration goals). Each indication-specific EMBARK treatment manual provides guidance on which integration goals to consider with the participant based on the specific treatment events that arose during the medicine session (see Figure 3 for an example). This section further details how EMBARK’s eclectic, six-domain approach comes together into a unified approach across the three phases of treatment.

#### Preparation Sessions

All EMBARK protocols developed so far have included three preparation sessions leading up to each medicine session, though this may change if called for by the indication under study. The therapists’ general aims for this phase have included building rapport and trust, learning about the participant’s experience of their mental health challenges, explaining basic elements of PAP treatment and what to expect from the medicine session, providing preparatory instructions about diet and aftercare, and responding to participant questions about PAP treatment.

Therapists are also given domain-specific tasks in the preparation phase (see Figure 1), which may vary across protocols in response to the specific proposed mechanisms of change for the clinical indication under study. These tasks lay the groundwork for the participant to receive benefit within whichever of the six domains become important for them during the medicine and integration phases. It is considered important to prepare every participant for potential benefit across all six domains because EMBARK therapists make no determination before the medicine session about which domains may or may not become relevant later in treatment, despite any prediction or wish held by them or the participant during this phase. Even if a participant frames their MDD in, for example, primarily spiritual terms and expresses a desire to address their symptoms within a spiritual framing, it is still very possible that treatment benefits will occur for them in other domains, in addition to or instead of the “E” domain, if they are properly prepared. During preparation, the participant’s lived experience of their symptoms and their functional significance are assessed, but no attempt is made by therapists to use this information to focus the participant’s treatment on a specific subset of psychedelic-induced phenomena during the medicine session. However, this information is taken into consideration later during the integration phase when deciding what integration goals would be most supportive.

Since it is unknown during the preparation phase which domains and domain-associated mechanisms of change will be most relevant to the specific participant, therapists’ role in this phase tends to be more standardized than it is in later phases. However, while preparation tasks are fixed in their intent, therapists are invited to bring in interventions from their preferred clinical orientation(s), as long as they conform to the guidelines that ensure that the intended utility of each task is conferred to the participant. For example, therapists tasked with teaching basic mindfulness skills to a participant may do so using mindfulness-based tools from ACT, DBT, other mindfulness-based EBTs, or a meditative spiritual tradition, provided that the tool meets the following criteria set forth in the EMBARK manual: (1) it invites the participant to cultivate a receptive, attentive state, (2) does not contain elements that could potentially clash with a participant’s religious beliefs, (3) and abides by trauma-informed practices detailed in the manual. The manual also provides example interventions for therapists who do not have relevant expertise to bring in for any given task.

Ethical considerations based on the four care cornerstones are woven throughout the guidelines set forth for therapist interventions. For example, the guidelines for the discussion of therapist-participant dynamics requires that it include an exploration of cultural dynamics, in line with the cornerstone of culturally competent care.

#### Medicine Sessions

At the outset of a medicine session, the therapists maintain an agnosticism about which benefits and which domains will ultimately become most salient for a participant, much as they did in the preparation phase. The pre-dosing therapist tasks (see Figure 1) thus serve a similar purpose to the tasks in the preparation phase in that they set the stage for phenomena in any domain to arise and bring the potential for benefit. These stage-setting tasks are woven together into a suggested pre-dosing agenda for the medicine phase that includes a collaborative ritual (E), a check-in about intentions (K), and a brief somatic awareness practice (B). The six EMBARK domains thus continue to serve therapists as a conceptual frame for laying the groundwork for a broad variety of pro-therapeutic outcomes.

However, once a psychedelic medicine is administered, the therapists’ role becomes more responsive to the specific situation and less about a domain-agnostic approach to preparation. At this point, the course of a participant’s treatment starts to come into focus, and specific EMBARK domains present themselves as more pertinent to the participant’s experience of the medicine. EMBARK’s six-domain framework comes to serve therapists in a new way as a practical rubric for helping them to characterize in-session events and determine what domain-specific interventions these events might call for. For example, if they observe the participant experiencing what they identify as a somatic trauma process, they can recall the training they received in the Body aware domain, and apply the interventions associated with it. Support in identifying and responding to these events is provided in the EMBARK training program (see later section) and in each indication-specific EMBARK manual.

The value of organizing the preparation work by domains becomes clear during this phase, as many of the responsive interventions applied in a particular domain will draw upon work that was conducted with the participant during the preparation phase in that same domain. For example, a reminder to “move toward” challenging emotional material will draw from preparatory psychoeducation provided in the Affective-cognitive domain, or a reminder to use a somatic self-soothing technique in response to intense somatic events will rely on what was taught during preparation in the Body aware domain. Therapists’ incorporation of their own favored interventions during the preparation phase also ensures that they will be working squarely within their competence during this more unpredictable phase as well.

#### Integration Sessions

At this point, the therapists and participant are likely to have a strong sense of the most beneficial direction for a participant’s continued therapeutic progress. During the first debrief session, all parties collaboratively determine what treatment goals might be best to work toward in integration. This process entails (1) debriefing and supporting the participant’s sense of what transpired in the medicine session, (2) relating material that arose in the medicine session to their symptoms and treatment goals, (3) collaboratively identifying new attitudes, beliefs, behaviors, values, or other subjective shifts that may contribute to symptom reduction, and (4) planning post-treatment changes that may support and sustain this outcome.

A set of suggested integration goals are provided in each indication-specific manual, along with guidelines and suggestions for working toward each of these goals. These goals are organized by domain to provide continuity with events that arose in the medicine session. Together, therapists and participants choose a subset of these goals, or develop their own, as long as they are based on a clear clinical rationale. This selection process is guided primarily by what transpired in the medicine session using a tool provided in each EMBARK manual (see Figure 3). For instance, a participant who had an experience that they identify as spiritual may benefit from support in advancing their spiritual self-development or spiritual practices (E), or a participant who had a strong emotional opening might be best served by continued processing and reflection that may lead to revised core beliefs or a sustained movement away from emotional avoidance (A). However, the selection process is also informed by the participant’s previously stated intentions for treatment and the functional meanings of their symptoms. For instance, if a depressed participant initially framed their suffering in terms of loneliness and set an intention of understanding their isolation, it might benefit them to consider goals in the Relational domain, even if nothing observable transpired in that domain during the medicine session.

All integration goals are framed in terms of three possible spheres of change: individual behavior, personal context, and broader context. Individual behavior changes may include stopping a maladaptive behavior, changing an old behavior, or adopting a new practice. Personal context changes may include updates to social, vocational, or physical contexts that support treatment benefit, such as moving away from a social circle that encourages problem drinking or moving into a vocational field more congruent with one’s revised personal values. The broader context refers to structural, cultural, or economic conditions that have real, mental health consequences for the individual. Participants who decide that change at this level would be supportive for them may benefit from taking collective action (e.g., community activism, labor organizing) that addresses broader conditions in a way that feels congruent with their revised values or sense of self and/or serve as a form of socialization or behavioral activation. This may have acute benefit for them (Fang et al., 2018; Hayhurst et al., 2019; Hui et al., 2020) while also contributing to the amelioration the conditions that had engendered or exacerbated their distress in the first place (e.g., exploitative work conditions). Participants and therapists determine together which of the three spheres might be appropriate foci for post-treatment changes that support participant wellbeing.

The number of integration sessions is left to the discretion of the group employing EMBARK, though all EMBARK manuals written to date have included a standard of three integration sessions per medicine session. It is suggested that, whatever the intended number may be, therapists and participants both use their judgment to determine if additional sessions would be supportive of the participant’s wellbeing and to undergo these sessions whenever possible. Otherwise, therapists working within the EMBARK approach are required to be prepared to make appropriate outside referrals if a participant requires ongoing therapeutic support. While EMBARK was designed to support short-term PAP interventions in clinical trial settings, its authors recognize the crucial importance of aftercare (Watts, 2022) and urge any organization adopting the EMBARK model to recognize the intensive nature of PAP treatment and ensure that participants’ wellbeing is properly supported after their participation in the trial.

## Cushion Presence

The quality of presence provided by clinicians during medicine sessions is considered to be a central element of PAP, due in large part to the heightened sensitivity of a participant in that setting (Johnson et al., 2008; Phelps, 2017; Thal et al., 2021, 2022). In EMBARK, therapists are encouraged to adopt a presence that reflects attributes represented by the acronym “CUSHION.” These attributes include Calm, Unhurried, Supportive, Human, Impeccably boundaried, Openhearted, and Non-judgmental. Training in the EMBARK approach encourages therapists to engage in self-directed practices of their choice (e.g., mindfulness, grounding exercises) in order to cultivate these attributes in themselves prior to engaging with a participant and ongoingly throughout their work as PAP therapists. EMBARK holds that one’s own internal work as a therapist is central to delivering the therapy in a manner that aligns with the four care cornerstones of the program and is inextricably linked with good outcomes.

## Growth Areas for EMBARK

The authors of EMBARK have intended its development to be an ongoing, iterative process. Some areas in which further development would be helpful have been identified. For example, while EMBARK’s delineation of six domains serves as a helpful rubric to add structure to the protean nature of treatment with psychedelic medicines, it also introduces a broad set of required competencies that may seem daunting to therapists. In essence, EMBARK asks PAP therapists to simultaneously consider six possible avenues of healing during treatment and to be able to respond competently to events that pertain to all of them. In response to this concern, aspects of EMBARK, such as its integrative agendas (Figure 2) and therapist checklists (Figure 3), were designed to help therapists develop a sense of where to direct their attention at all points during treatment. However, the process of streamlining EMBARK is still underway, and further supports for therapists will continue to be incorporated.

EMBARK’s inherently eclectic design may also leave it prone to some of the same shortcomings attributed earlier to basic support models (see Table 2), particularly in the operationalization of interventions used in a clinical trial. The breadth of possible interventions, coupled with the allowance that therapists can draw from their favored clinical orientation(s), may complicate the creation of adherence criteria. The authors feel that the intervention guidelines presented in the manual for therapist interventions can serve as a strong foundation for drafting clear, meaningful criteria, even if the specific interventions they allow for are diverse. In future iterations of EMBARK, these interventions guidelines will continue to be refined with an eye toward their enhanced contribution to the process of developing adherence criteria.

EMBARK also shares a limitation with EBT-inclusive approaches in that it is unclear if the efficacy of the EBTs it incorporates is preserved by the way in which they are brought in. Ultimately, this is a clinical question to be answered by further research, perhaps in a head-to-head trial that compares EMBARK to an EBT-inclusive approach that relies on a single EBT applied in a way that hews closer to its original, empirically validated form.

Finally, EMBARK’s situatedness within a Western medical framework and its prioritization of EBTs limits its ability to draw from other rich sources of knowledge on the use of psychedelic medicines, including indigenous approaches. EMBARK’s authors made this scope decision so that the model could be maximally useful in clinical trials that assess whether psychedelic medicines can be efficacious within the institutions of Western psychiatry and psychotherapy and their associated conceptualizations of healing. In making such a choice, EMBARK lost the ability to directly incorporate a great deal of wisdom from other legitimate models of how psychedelics can be of benefit to humanity. It is the authors’ hope that others with greater expertise in non-medical approaches will find value in putting them in dialogue with EMBARK.

## EMBARK Training

The EMBARK training approach described in this article is intended to train PAP clinical trial facilitators to basic competency in supporting participant benefit in a clinical trial as described previously. It consists of training in four areas: specific training modules in the EMBARK clinical domains and care cornerstones, training in specific skills required for working in a clinical trial, indication-specific training, and experiential training in an expanded state of consciousness. Taken together, the total length of training is 60 h and can include a several-day experiential retreat, if desired. Other aspects of training, such as the specifics of supervision and peer consultation or whether trainees are required to be licensed or license-eligible psychotherapists prior to EMBARK training, are left to the discretion of the group adapting EMBARK in consideration of the specific needs of their participants and trainees. In its trial-specific form, as presented here, EMBARK training is not intended to provide facilitators with the competencies required to become a state license-eligible psychotherapist. However, we suggest that EMBARK’s six domains and four cornerstones could be used as the basis for developing a more in-depth PAP facilitator training program in another setting that also provides elements of general competency in psychotherapy.

### Training in Domains and Cornerstones

EMBARK therapists receive specific training in the knowledge, skills, and awareness required to support benefit in the six clinical domains. Trainees also receive specific training in each care cornerstone to ensure that they can provide care in line with the full breadth of their ethical commitment to the participants under their care. These 10 modules use a flip class design that asks trainees to complete readings and watch a 1-h prerecorded video before engaging in a live 2-h training session. An additional introductory module that precedes these presents an overview of the EMBARK approach to orient therapists to the model. Once the one introductory and 10 core modules are complete, trainees undergo a final integrative module that draws all that they learned into the integrated treatment approach found in the manual for that trial. Throughout each clinical trial, EMBARK therapists are also required to engage in ongoing clinical supervision and regular peer-led consultation groups.

Organizing PAP training around the six domains and four care cornerstones allows for the recruitment of 10 different faculty members who are experts in each of these areas. This presents an additional advantage over a single-teacher approach in that trainees will receive a variety of perspectives on how to conduct PAP treatment. This model also ensures that each teacher presents material from within a domain that speaks to their particular area of expertise, rather than asking one teacher to speak to all domains. For example, Jeffrey Guss, MD, an expert in relational psychoanalysis, taught in the Relational domain for the inaugural EMBARK training, while Adele Lafrance, Ph.D., an expert in EFT, taught in the Affective-cognitive domain.

### Indication-Specific Training

As a transdiagnostic model of PAP, EMBARK is being adapted for different indications, including MDD, alcohol use disorder, anxiety disorders, etc. For each clinical trial that applies EMBARK to the PAP treatment of a specific indication, therapists undergo an additional training module on the particularities of working with that indication. This module is led by a faculty member who is a recognized expert in the treatment of the indication under study. Additionally, it is recommended that each clinical trial that employs EMBARK invite clinical supervisors with expertise pertinent to the indication under study.

### Clinical Trial Training

When the EMBARK approach is used in a research setting, study therapists are also required to undergo training in skills relevant to conducting PAP in the context of a clinical trial. Currently, this includes four 2-h modules that cover the basics of clinical research, ethics and consent in clinical trials, roles and responsibilities in clinical trial documentation, and responding to adverse events. It also includes self-paced training in the International Conference on Harmonization Good Clinical Practice (ICH-GCP), Basic Life Support (BLS), the use of the Columbia Suicide Severity Rating Scale (C-SSRS), and certification in the Collaborative Institutional Training Initiative (CITI) Program.

### Experiential Training

It has been suggested that personal experiences with altered states of consciousness may improve PAP therapists’ ability to support participants by giving them an enhanced understanding of the subjective experiences their participants may undergo in a psychedelic medicine session (Nielson, 2021). It may also give therapists a firsthand sense of the vulnerability and suggestibility engendered by psychedelic medicines in a way that could help them minimize relational harm to participants. As such, one organization funding and administering clinical trials assessing the efficacy of MDMA in the treatment of PTSD has obtained permission to conduct a healthy volunteer trial that allows trained study therapists to undergo a medicine session as a participant (MAPS, 2020). However, providing an experiential training opportunity has proven challenging for other research groups due to legal factors, stigma around drug use, and this practice’s affront to the institutional logic of psychiatry, which does not value or require personal experience with medications administered to patients (Nielson and Guss, 2018). This practice has also come under scrutiny for potentially introducing a source of bias into research in that therapists who take a psychedelic medicine for the first time in preparation for working on a clinical trial may develop an inflated sense of the drug’s capacity to heal, which could have implications for research blinding and objectivity in the presentation of results (Anderson et al., 2020). Furthermore, investigator self-disclosures about personal experience with psychedelic drugs has been found to degrade perceptions of their integrity as a researcher held by psychedelic-naïve observers (Forstmann and Sagioglou, 2021). Given this set of important considerations, clinical research groups who employ the EMBARK approach are free to decide whether they will include an experiential training component and whether this component will include an opportunity to take a psychedelic drug or an alternative practice (e.g., breathwork).

## EMBARK in Action

Although EMBARK has been trademarked by Cybin Inc., it has been made freely available to any group interested in adopting it for their own purposes, research-related or otherwise. As noted earlier, the EMBARK approach is adaptable to almost any PAP clinical trial that uses a psychedelic medicine to treat a clinical indication. The approach is also flexible enough to serve as the basis for a more in-depth PAP therapist training that supports trainees in becoming more skilled PAP providers. For instance, EMBARK will be used to train the therapists who staff a low-cost/no-cost psychedelic treatment clinic under development at Lenox Hill Hospital with the support of a grant from Cybin.

At Cybin, upcoming clinical trials using the proprietary CYB003 and CYB004 formulations will use the EMBARK approach to target MDD, alcohol use disorder (AUD), and anxiety disorders. Treatment manuals have already been written for MDD and AUD, which have tailored the EMBARK approach to these indications. For example, the AUD manual incorporates a strong focus on relapse prevention in its treatment goals and sets intervention guidelines based on the practices of several AUD-specific EBTs, such as MBRP (Bowen et al., 2009) and MI (Riper et al., 2014). Despite the considerable differences between AUD, MDD, and anxiety-related disorders—as well as the differences between CYB003 and CYB004—the EMBARK model’s flexible, open architecture has been found to be meaningfully applicable to developing therapeutic approaches for all studies described here.

EMBARK is intended to continually evolve by way of collaborative input from research groups outside of Cybin that adapt the model for their own purposes. For instance, the collaboration between the current authors and Dr. Back on adapting EMBARK for his clinical trial, discussed in the introduction, led to enduring changes in the way EMBARK is taught and presented to therapists. Some of Dr. Back’s innovations have become standard in other EMBARK manuals, such as the inclusion of an integration guidance checklist (see Figure 3) and “cheat sheets” that provide a concise summary of therapist tasks, clarified presentation of therapist tasks throughout the manual, and a reorganized section on managing challenging medicine session events. Dr. Back also co-developed the EMBARK training program his study facilitators underwent, which will serve as the template for future trainings. Additionally, Ladybird Morgan, RN, MSW, a lead facilitator for this study, authored a section on diversity and inclusivity that is now found in all EMBARK manuals. As more outside groups shape EMBARK to their own needs, we hope that its open-source model will be further developed and strengthened through a continual process of collaborative peer review.

## Conclusion

The field of PAP research may be seen as entering its own critical period of social learning. It has begun to engage with questions of how to ensconce itself within the lineup of more established psychotherapeutic approaches. To do so, it has wisely looked outside itself to the clinical frameworks offered by leading EBTs. This cross-pollination will be helpful if and when PAP scales up, as it makes PAP more accessible to the thousands of therapists seeking to participate in the process. However, the field must not lose sight of the irreducible differences between PAP and the EBTs it borrows from. It should carefully consider the pros and cons of how it interfaces with existing psychological knowledge, lest it lose something essential.

We offer the malleable, plug-and-play structure of the EMBARK model as a useful staging ground for any attempt at the judicious creation of a syncretic treatment approach. It is presented here as an adaptable frame for PAP that synthesizes current knowledge in the field in the service of supporting our evolving collective understanding of the spectrum of ways in which psychedelic medicines may bring novel, beneficent possibilities to participants and societies.

## Data Availability Statement

The original contributions presented in the study are included in the article/supplementary material, further inquiries can be directed to the corresponding author/s.

## Author Contributions

WB and AB were main contributors to the development of the EMBARK model presented in the manuscript, collaborated on the organization of the manuscript and the ideas set forth therein. WB wrote the initial draft of the manuscript. AB provided edits and input toward the final draft. Both authors contributed to the article and approved the submitted version.

## Conflict of Interest

Both authors have received financial compensation from Cybin, Inc., during the time in which EMBARK was developed and when this article was written. WB was a paid consultant at Cybin, and AB was Cybin’s Chief Clinical Officer. In the last 3 years, AB has also received financial compensation for psychedelic research-related activities from Yale University, NYU School of Medicine, MAPS Public Benefit Corporation, Heffter Research Institute, Synthesis Institute, Chacruna Institute, and Adelia Therapeutics and has filed patents on the use of psychedelic compounds for the treatment of psychiatric indications.

## Publisher’s Note

All claims expressed in this article are solely those of the authors and do not necessarily represent those of their affiliated organizations, or those of the publisher, the editors and the reviewers. Any product that may be evaluated in this article, or claim that may be made by its manufacturer, is not guaranteed or endorsed by the publisher.
